# Cholera Outbreak in Grande Comore: 1998–1999

**DOI:** 10.4269/ajtmh.15-0397

**Published:** 2016-01-06

**Authors:** Christopher Troeger, Jean Gaudart, Romain Truillet, Kankoe Sallah, Dennis L. Chao, Renaud Piarroux

**Affiliations:** Institute for Health Metrics and Evaluation, University of Washington, Seattle, Washington; Sciences Economiques and Sociales de la Santé and Traitement de l'Information Médicale, Aix-Marseille University, Marseille, France; Centre d'Investigation Clinique–Centre de Pharmacologie Clinique et d'Evaluation Thérapeutiques, Assistance Publique Hôpitaux de Marseille, Marseille, France; Vaccine and Infectious Disease Division, Fred Hutchinson Cancer Research Center, Seattle, Washington; Aix-Marseille University, Marseilles, France

## Abstract

In 1998, a cholera epidemic in east Africa reached the Comoros Islands, an archipelago in the Mozambique Channel that had not reported a cholera case for more than 20 years. In just a little over 1 year (between January 1998 and March 1999), Grande Comore, the largest island in the Union of the Comoros, reported 7,851 cases of cholera, about 3% of the population. Using case reports and field observations during the medical response, we describe the epidemiology of the 1998–1999 cholera epidemic in Grande Comore. Outbreaks of infectious diseases on islands provide a unique opportunity to study transmission dynamics in a nearly closed population, and they may serve as stepping-stones for human pathogens to cross unpopulated expanses of ocean.

## Introduction

The seventh pandemic of cholera reached the African continent in 1970 and spread widely in the subsequent years.^1^ In 1975, the Union of the Comoros, a small nation composed of a volcanic island archipelago in the Indian Ocean on the northern end of the Mozambique Channel between mainland Africa and Madagascar, reported its first cases of cholera.^2^ After this outbreak, no cases were reported in the Comoros for over 20 years—until 1998.^2^ Between early January 1998 and late March 1999, Grande Comore Island, the largest island in the Union of the Comoros, experienced a cholera epidemic affecting nearly 8,000 people.^3^

Despite the magnitude of the 1998–1999 cholera outbreak in the Comoros Islands, little has been published on the epidemiology of cholera in Grande Comore. In addition, except for the ongoing cholera epidemic in the Caribbean Islands, few outbreaks on islands have been described in the literature. Using data collected during the outbreak and the senior author's (Renaud Piarroux) personal observations from his involvement in the outbreak response, this article describes the epidemiology of the 1998–1999 cholera outbreak in Grande Comore.

## Materials and Methods

### Outbreak data.

The French Ministry of Foreign Affairs organized cholera surveillance during the epidemic. A doctor within the Ministry provided cumulative cholera case counts at roughly weekly intervals, with some minor inconsistencies in reporting periods. Renaud Piarroux, at the time a member of *Médecins du Monde*, made field observations and provided the description of the medical response. The cholera surveillance occurred at the district and locality levels. There are seven districts in Grande Comore composed of 168 localities.

The population data for Grande Comore were obtained from a 1997 census report.^4^ Geographical coordinates of most localities are from Google Maps (http://www.google.com/maps), with the locations of the remaining localities obtained from the National Geospatial Intelligence Agency (http://earth-info.nga.mil/gns/html/) and Index Mundi (http://www.indexmundi.com/zp/cn/). Highway location data are from OpenStreetMap (http://www.openstreetmap.org). Driving distance data were obtained using the Google Maps Distance Matrix API (https://developers.google.com/maps/documentation/distancematrix/).

### Spatial analyses.

To assess spatial autocorrelation of cholera incidence by locality, we used the Moran's I statistic and the local indicator of spatial autocorrelation (sometimes called the local Moran's I test) and used Euclidean and road network distances to define neighboring localities.^5^,^6^ SaTScan™ software was used to identify spatiotemporal clusters of cholera cases.^7^–^11^

### Force of invasion.

We define the date of the first reported cholera case in a locality as “invasion.” We assume that for a given cholera-free location, the risk of a new cholera case appearing is related to the number of cases in other locations weighted the inverse of their distances. We call this the “force of invasion.” Here, we assume that the force of invasion is equal to the number of nearby cases divided by a function of distance to the disease-free location. We use this measure to predict for a given location l that it will report cases at time *t*, given that this location has not seen cases earlier. Because reports were not completely regular in time, we interpolated the daily number of reported cases when estimating the number of cases occurring over a time interval.

We defined the force of invasion based on a gravity law from Xia and others^12^:

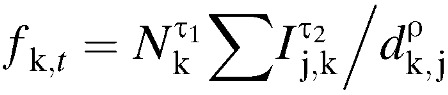
where *f* is the force of invasion at location k at time *t*, *N*_k_ is the number of susceptible people (population size) in location k, *d*_k,j_ is the distance between locations k and j, ρ exponentiates the distance, and τ_1_ and τ_2_ exponentiate the susceptible or infected population sizes. Here, we define *I*_j,t_ to be the number of reported cholera cases that were reported at location j between 1 and 2 weeks before *t*. We used a receiver operating characteristic curve to find an “optimal” threshold for using this metric as a predictor of invasion. We calculated areas under the curve (AUCs) for different values of τ_1_, τ_2_, and ρ. We explored the parameter space using a grid search at a resolution of 0.1 for the three parameters (ρ = 0 … 2.0, τ_1_ = 0 … 1.2, τ_2_ = 0 … 1.2). Previous studies had found ρ could be as high as 3 in some settings, but the limitations in mobility in Grande Comore would make it likely that ρ would be lower. We anticipated that τ_1_ and τ_2_ would be less than 1, since it seemed likely that the force of invasion would have a linear or sublinear relationship with the number of infected people or the population size.

## Results

Cholera was introduced to Grande Comore in late 1997 at a time when particularly large outbreaks were hitting Tanzania and Mozambique, two African countries bordering Indian Ocean with coasts less than 200 miles from the Comoros.^13^,^14^ The first case was reported in the village of Mbéni on the northeast coast of the island in January 1998.^15^ The WHO was notified of a possible cholera outbreak on January 19 that was later confirmed to be *Vibrio cholerae* O1 El Tor.^15^ The deaths of two cholera patients transferred to a hospital in Moroni, the capital, in early February may have brought a greater sense of urgency to respond to the epidemic.^3^ Field observations suggested nosocomial cholera transmission during the early stages of the epidemic in hospitals in Moroni.^3^

**Figure 1.**
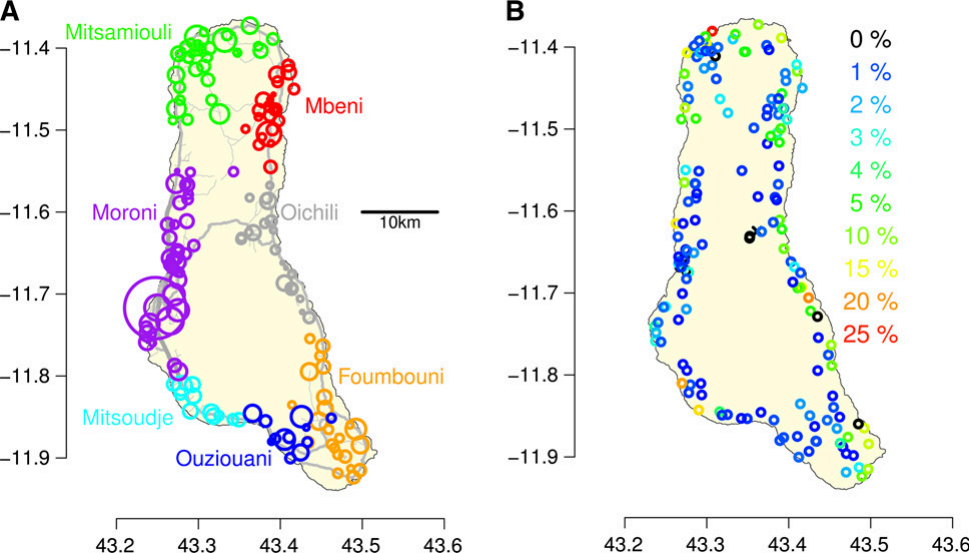
Maps of Grande Comore. (**A**) Localities of Grande Comore are marked with circles color coded by district, with each circle's area proportional to the population. Major roads are drawn in gray, with thicker lines for primary and secondary highways and thinner lines for smaller roads. Degrees latitude and longitude are indicated on the axes. (**B**) Map of final reported cholera incidence. The color of the circles is based on the final reported suspected cholera incidence of each locality, from blue for low incidence to red for high (up to 25%). Black Xs indicate localities that did not report any cholera cases.

Cholera initially appeared to spread by road across the island of Grande Comore, with first striking Mbéni and Moroni districts, then spreading to neighboring areas. A main road circles the island, a few smaller roads traverse the center, and the active volcano Karthala hinders travel across the middle of the island (Figure 1A

).

**Figure 2.**
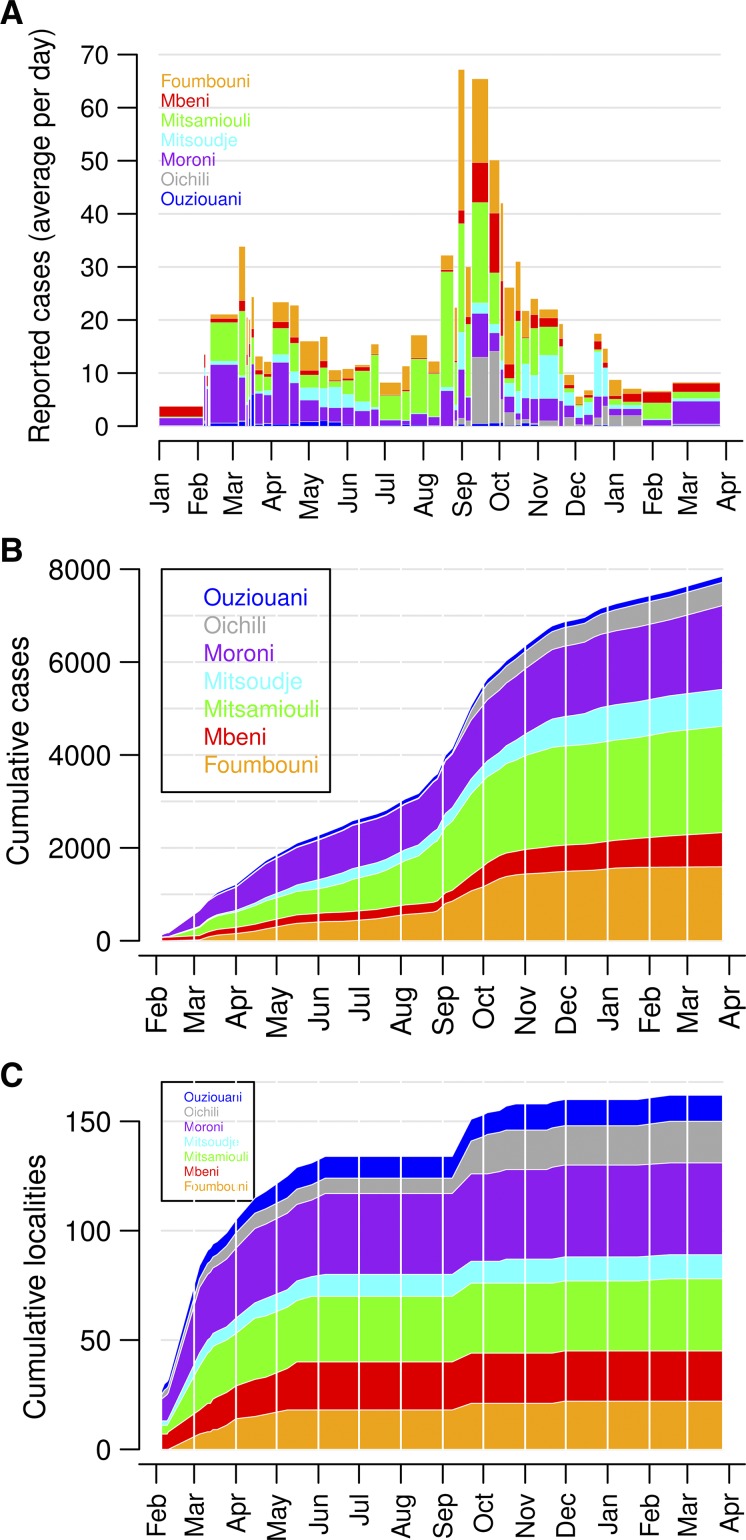
Cholera epidemic curves by district. (**A**) Reported suspected cholera cases. Each bar represents one reporting interval, with the right of the bar on the actual reporting date and the left of the bar at the date of the previous report, or January 1, 1998 in the case of the first report. The height of the bars is the number of cases in each report divided by the days since the last report and estimate of the average number of cases reported per day. Therefore, the area of each rectangle is proportional to the number of cases in a district in each report. Note that the reports are not evenly spaced in time. (**B**) Cumulative reported suspected cases by district from January or February 1998 to April 1999. The *x* axis is exact report date (not converted to weekly intervals). (**C**) The cumulative number of localities in each district (color coded) that have reported cholera cases.

The number of reported cases rose sharply in February 1998 (Figure 2

). By the first week of March, cholera had been reported in six of the seven districts on the island (and in nearly half of the localities as seen in Figure 2C). The epidemic reached its first peak in March after cholera invaded the southern districts Ouziouani and Foumbouni (Figure 2). The first hospitalized case in the southeastern district of Foumbouni was a cook from the northern part of the island, visiting the village of Ouroveni to help prepare a grand marriage feast in early March 1998.^3^ A few days later, while a few sporadic cases were being treated in the nearby Foumbouni hospital, a sudden outbreak started in Ouroveni with 104 people hospitalized during the week March 10–17. A rapid field investigation performed by a Médecins du Monde team indicated that all infected people hospitalized on the first day of the outbreak had drunk untreated water drawn from a well located a few hundred meters from the village (R. Piarroux, personal communication). This well, which was not protected by a curb, was regularly used by one of the patients who presented cholera symptoms just after the grand marriage collective meal.

By May and June, the epidemic appeared to be waning (Figure 2A) and funding for the cholera response ceased in July. However, the dry season, “kusi,” began in May. There are no rivers in Grande Comore, and so aside from rainwater collection cisterns, the only sources of water are brackish “piscines” that are filled by the rise of the water table, pushed up by the tide. These piscines, also called “marigots,” are used for many purposes including washing clothes, dishes, washing oneself, and sometimes for defecation. Water scarcity forces Comorians to use piscines and other unimproved sources for water.

The second peak in cholera activity, in August and September 1998, was much larger than the first, and cases were reported in all seven districts on the island (Figure 2). Grand marriage festivals, implicated in the initial spread to the southern districts of the island, were concentrated in August.^16^

By March 26, 1999, the end of the epidemic, there were 7,851 reported cases of cholera in Grande Comore (Table 1, Figure 2B). The incidence of cholera during the 1998–1999 epidemic was 30.4 reported cases per 1,000 population (Table 1). The attack rate in the capital city of Moroni, with a population of about 35,000, of 12.7/1,000 was lower than average. In Moroni, most residents had access to piped water, unlike much of the rest of the island. Overall, 163 of 169 localities reported suspected cholera cases (Figures 1C and 2C). Many localities in districts on the north (Mitsamiouli), east (Mbeni and Oichili), and south (Foumboni) coasts had high attack rates (above 5%). Most localities in Oichili District on the island's eastern side did not report cholera cases until September 1998 (Figure 2C).

**Figure 3.**
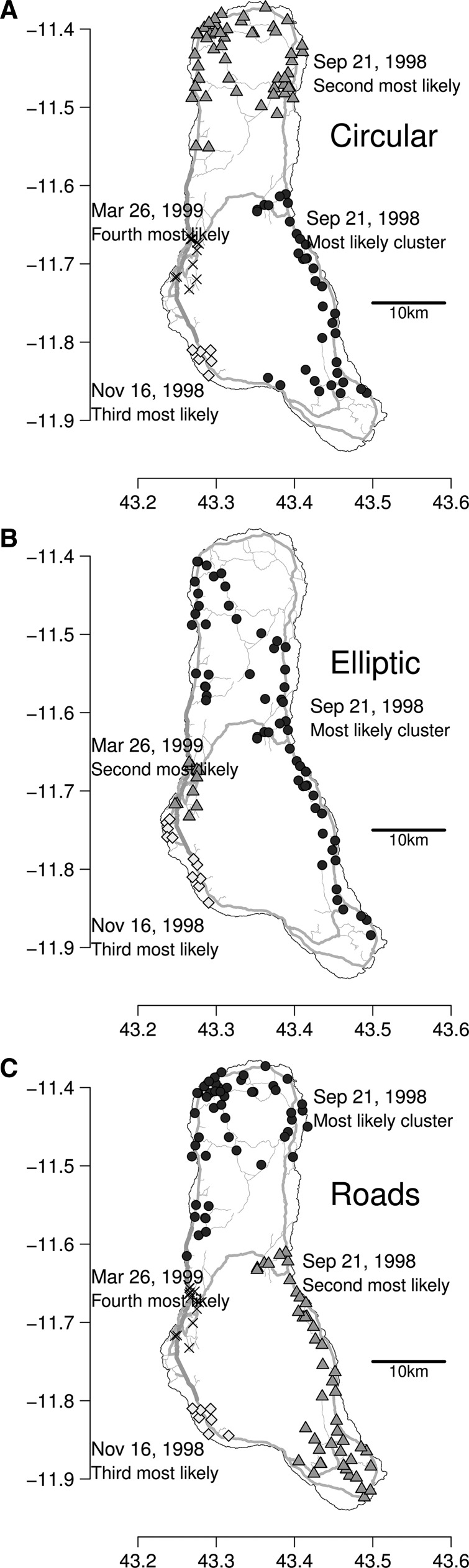
The most likely spatiotemporal clusters of cholera cases. SaTScan software was used to identify spatiotemporal clusters of reported suspected cases. The maximum allowable cluster size was 25% of the total population of Grande Comore. The date and relative likeliness of the clusters are indicated. (**A**) Assuming circular clusters, the relative risks in each of the four clusters were 128.72, 85.41, 148.09, and 33.51. (**B**) Assuming elliptical clusters, the relative risks in the three clusters were 131.62, 33.75, and 84.21. (**C**) Clusters were identified as localities within a given driving distance from the center of the cluster. The relative risks in the four clusters were 95.99, 107.66, 115.04, and 32.75.

We identified spatiotemporal clusters of reported suspected cholera cases using SaTScan software (Figure 3

). The most likely (and largest) cluster occurred on September 21, 1998, and covered the northern and eastern sides of the island. The second most likely cluster occurred on November 16, 1998, in Mitsoudje District. The third most likely cluster occurred on March 26, 1999, in Moroni district. These results were robust to assumptions about the shapes of the clusters, but the maximum identifiable cluster size was set to 25% of the island's population (rather than the default 50%) to prevent September 21 cluster from encompassing too many localities, particularly when circular clusters were used.

**Figure 4.**
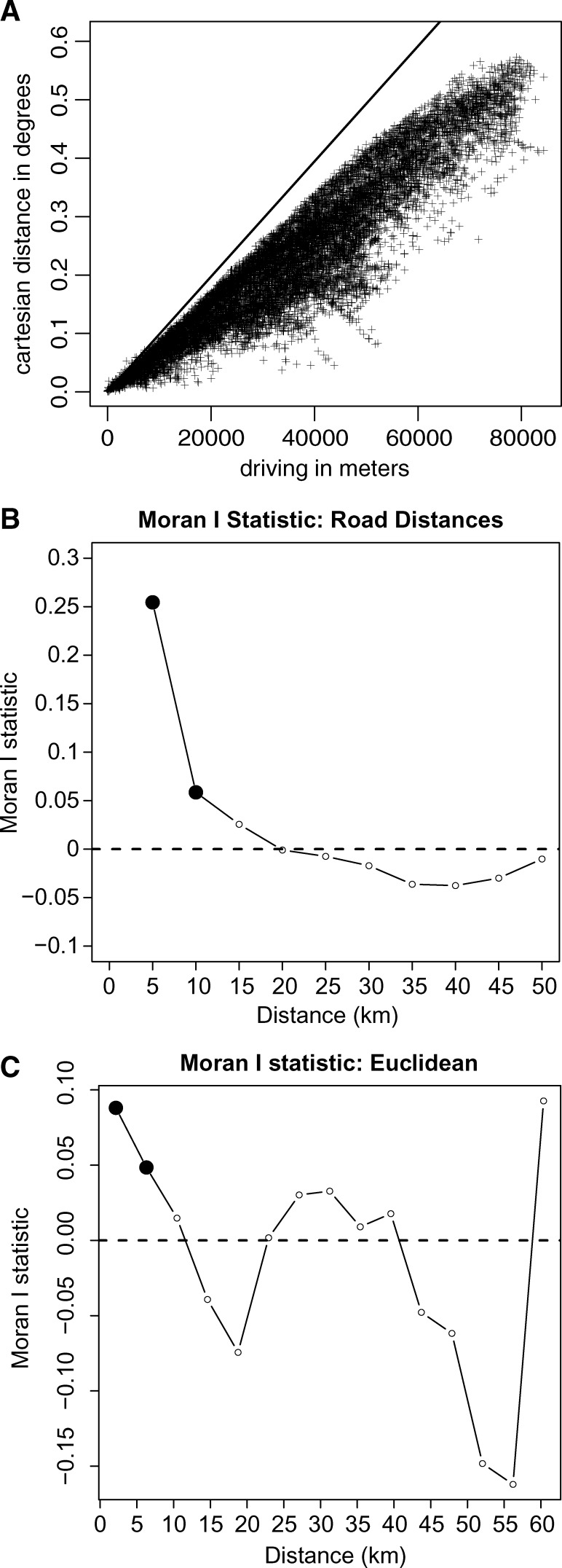
Driving distance and Euclidean distance among localities in Grande Comore. (**A**) Driving vs. Euclidean distance between all pairs of localities. The diagonal line assumes that 1° equals 109 km (an approximation for 11.6° S latitude). A comparison of global Moran's I scores for spatial autocorrelation of final attack rates when neighbors were defined using (**B**) driving distances and (**C**) Euclidean distances. Filled circles represent Moran's I scores that are statistically significant at *P* < 0.05. The distance classes for the Euclidean distance in (**C**) assume that 1° equals 109 km.

Localities that were near each other by road had correlated final reported incidence (global Moran I = 0.25, *P* = 0.01), and incidence was significantly spatially autocorrelated up to 10 km away (Figure 4

). Defining spatial neighbors by road distance was a better indicator of spatial autocorrelation than by Euclidean distances.

**Figure 5.**
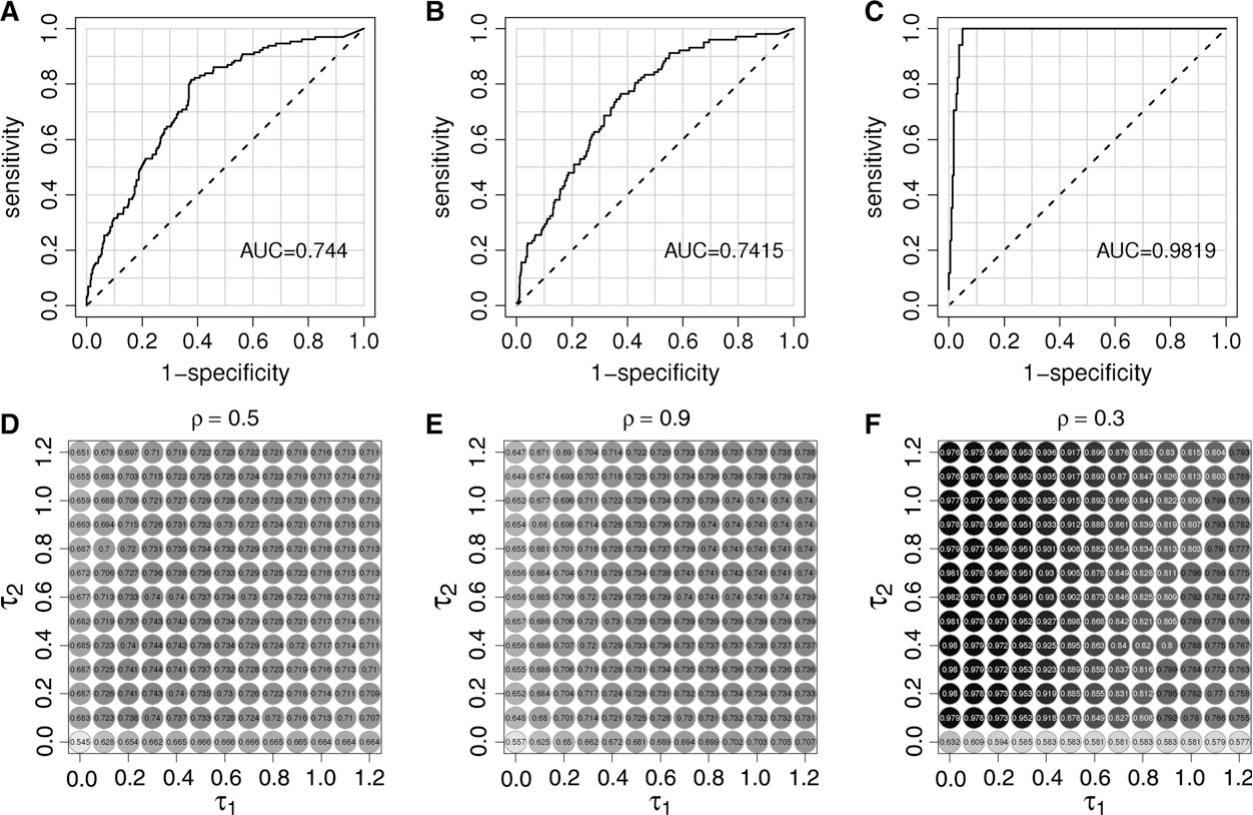
Force of invasion. The force of invasion for localities that had not reported cholera cases was associated with an increased probability of the arrival of cholera. The force of invasion is based on the number of cases reported between 1 and 2 weeks ago and the driving distance to those cases. The receiver operating curves (ROCs) and areas under the curves (AUCs) for using the force of invasion to predict the first report of cholera cases of a new locality for (**A**) the entire cholera epidemic, (**B**) only reports before July 31, 1998, and (**C**) only reports from August to September 1998. These curves are for the free parameters (i.e., ρ, τ_1_, and τ_2_) associated with the highest AUCs. Panels **D**–**F** show the AUCs for different combinations of τ_1_ and τ_2_ are shown for the same periods and values of ρ as panels **A**–**C**. AUCs are printed on the plots in dark circles when the AUCs are high, in lighter circles for low AUCs.

We explored the invasion of cholera-free localities based on the number and distance of recently reported cases in other localities. We formalized this notion as the force of invasion, as described in the section Materials and Methods. We found only modest predictive value if this method was applied to the entire duration of the outbreak, with an AUC of 0.74 when ρ = 0.5, τ_1_ = 0.3, and τ_2_ = 0.3 (Figure 5A and D

). We hypothesized that the transmission of cholera was qualitatively different between the early and late phases of the epidemic. When we only consider reports from the early phase of the epidemic (February–July 1998), the method only had moderate success in predicting cholera invasion (Figure 5B and E; maximum AUC of 0.74 when ρ = 0.9, τ_1_ = 0.9, and τ_2_ = 0.7). We found that during the early phase, the population size of a disease-free locality was correlated with the probability of invasion (AUCs were low when τ_1_ = 0). When we consider only reports from the second peak of activity (from August to September 1998), the predictability of invasion was much better (Figures 5C, 4F; maximum AUC of 0.98, at ρ = 0.3, τ_1_ = 0, and τ_2_ = 0.6, but with all AUCs ranging from 0.75 to 0.98). During the second peak of activity, the number of infected people in localities played a role in the invasion of uninfected localities (i.e., AUCs were low, poor when τ_2_ = 0.0), but the size of the susceptible population and the distance from infected locations did not appear to play a role (i.e., AUCs were high when τ_1_ = 0 or ρ = 0). In other words, the early phase of cholera seemed to preferentially strike larger communities, while the late phase seemed to invade all communities regardless of size and may be even distance from outbreaks.

## Discussion

In late 1997 or early 1998, cholera arrived in Grande Comore and spread quickly across the island. The Comoros had not seen cholera in over 20 years, so the population was highly susceptible. The epidemic spread through a combination of diffusion to neighboring regions by road and a few long-range jumps. In several cases, the medical response teams witnessed direct contamination of water sources, such as one that occurred just after a grand marriage festival, that were probably responsible for the introduction of cholera from geographically separated localities.^3^ The peak of grand marriage activity overlaps with holiday months in France; nearly 80,000 Comorians are estimated to live in Marseilles, and each year roughly 4,000–5,000 travel back to the Comoros to visit family or participate in grand marriage festivals.^17^ Members of the Comorian diaspora in France earn more money and are more likely to fund grand marriages during their holiday months. The increase in cholera cases during the month of August 1998 coincided with an unusually large number of grand marriages in part because Mawlid, the prophet Mohammed's birthday, fell in July and many marriages were postponed until August. The grand marriage festivals would again be associated with the largest cholera epidemic peak during the 2007 cholera epidemic in Grande Comore. Similar to the epidemic in 1998, the International Federation of Red Cross and Red Crescent Societies concluded “a sudden increase in suspected cases … during the first weekend of the month of August … [was due to] a lack of basic hygiene measures, poor supply of clean water and the epidemic having an increased impact as the annual festival of grand marriages.”^18^ Medical response teams met resistance in behavioral change campaigns and found that many Comorians continued to use untreated water either out of habit or lack of alternatives.^3^ The end of the epidemic may have been brought about by a combination of outbreak response measures, a return to rainwater collection, and widespread population immunity from exposure earlier in the epidemic.

In Grande Comore, there are no rivers or other flowing water to carry *V*. *cholerae* in the environment, so cholera's spatial spread is driven entirely by the movement of infected individuals. This hypothesis is supported by the fact that the driving distance between localities was a better predictor of similarity of final cholera incidence than Euclidean distance. It is not clear if these correlations are due to the spread of cholera between neighboring communities or the potential similarity of conditions in nearby locations. We found that the arrival of cholera in new locations was related to the distance of recently reported cholera cases. Larger susceptible localities tended to be affected more quickly than smaller ones. One might expect that the interaction between two localities would be inversely proportional to the square of their distance, but we found the best fits when the distances were raised to a power less than 1. This is likely because the actual topology of the road network was used instead of Euclidean distances. Ideally, the actual movement of people, rather than estimates using driving distances in a parametric model, should be used to predict the spread of cholera, as was done in a recent study of the cholera outbreak in Haiti.^19^

Unfortunately, sustained funding was not available for the targeted treatment of water supplies in cholera-affected villages (R. Piarroux, personal communication), and the epidemic surged during the months of August and September 1998, 8 months after cholera was first confirmed in Grande Comore. Recently, oral cholera vaccine (OCV) has become a practical cholera prevention and outbreak response option.^20^ A global stockpile of Shanchol, one of two WHO preapproved OCVs, was established for outbreak response. OCV from the stockpile has been successfully deployed around the world and vaccination campaigns covering tens of thousands of people took from 1 to 3 months to complete.^20^ If sufficient quantities of OCV had been available in 1998, there could have been sufficient time to reduce or even prevent the second, and larger, peak of cholera in August 1998.

The Comoros Islands could be a stepping-stone for cholera and other infectious diseases to travel from mainland Africa to other islands in the southwestern Indian Ocean and vice versa.^21^ Genetic sequences of samples of *V*. *cholerae* in the Comoros and in Madagascar suggest that travelers, possibly traders or fishermen, introduced cholera in the coastal regions on the northwest part of Madagascar from the Comoros Islands in February 1999.^22^ The epidemic in Madagascar caused nearly 46,000 reported cases and killed over 2,600 people. Therefore, mitigating or preventing epidemics in the Comoros could break the chains of transmission of infectious disease that traverse large expanses of unpopulated ocean.

## Figures and Tables

**Table 1:** Final reported cholera incidence by district

District	Cases	Population	Final incidence (per 1,000)
Foumbouni	1,533	29,405	52.1
Mbéni	880	25,543	34.5
Mistamiouli	2,500	44,817	55.8
Mitsoudje	485	19,460	24.9
Moroni	1,799	104,743	17.2
Oichili	523	16,538	31.6
Ouiziouani	131	17,997	7.3
Total	7,851	258,503	30.4
